# C-Phycocyanin Ameliorates Mitochondrial Fission and Fusion Dynamics in Ischemic Cardiomyocyte Damage

**DOI:** 10.3389/fphar.2019.00733

**Published:** 2019-06-28

**Authors:** Jinchao Gao, Lidong Zhao, Jinfeng Wang, Lihang Zhang, Dandan Zhou, Jinlong Qu, Hao Wang, Ming Yin, Jiang Hong, Wenjuan Zhao

**Affiliations:** ^1^Engineering Research Center of Cell & Therapeutic Antibody, Ministry of Education, School of Pharmacy, Shanghai Jiao Tong University, Shanghai, China; ^2^Department of Internal and Emergency Medicine, Shanghai General Hospital, Shanghai Jiao Tong University School of Medicine (Originally Named “Shanghai First People’ s Hospital”), Shanghai, China; ^3^College of Chemistry and Environmental Engineering, Shandong University of Science and Technology, Qingdao, China; ^4^Department of Emergency and Critical Care, Shanghai Changzheng Hospital, Second Military Medical University, Shanghai, China

**Keywords:** C-phycocyanin, mitochondrial dynamics, fission, fusion, apoptosis, cardiomyocytes, ischemia

## Abstract

Mitochondrial dysfunction is a predominant risk factor in ischemic heart disease, in which the imbalance of mitochondrial fusion and fission deteriorates mitochondrial function and might lead to cardiomyocyte death. C-phycocyanin (C-pc), an active component from blue-green algae, such as *Spirulina platensis*, has been reported to have anti-apoptosis and anti-oxidation functions. In this study, the effects of C-pc on mitochondrial dynamics of cardiomyocytes was examined using an oxygen–glucose deprivation/reoxygenation (OGD/R) model in H9c2 cells, an *in vitro* model to study the ischemia in the heart. Cell viability assay showed that C-pc dose-dependently reduced OGD/R-induced cell death. Intracellular reactive oxygen species production induced by OGD/R was decreased in C-pc-treated groups in a dose-dependent manner as well. H9c2 cells subjected to OGD/R showed excessive mitochondrial fission and diminished mitochondrial fusion. C-pc treatment significantly ameliorated unbalanced mitochondrial dynamics induced by OGD/R and regulated mitochondrial remodeling through inhibiting mitochondrial fission while promoting fusion. The enhanced expressions of dynamin 1-like protein and mitochondrial fission 1 protein induced by OGD/R were suppressed by C-pc, while the subdued expressions of mitochondrial fusion proteins mitofusins 1 and 2 and optic atrophy 1 induced by OGD/R increased in C-pc-treated groups. Triple immunofluorescence staining revealed that C-pc treatment reduced the recruitment of dynamin 1-like protein from cytoplasm to mitochondrial membranes. Furthermore, C-pc protected H9c2 cells against OGD/R-induced cytochrome c/apoptotic protease activating factor-1 intrinsic apoptosis and suppressed the phosphorylations of extracellular signal-regulated kinase and c-Jun N-terminal kinase. These results suggest that C-pc protects cardiomyocytes from ischemic damage by affecting mitochondrial fission and fusion dynamics and reducing apoptosis and, thus, may be of potential as a prophylactic or therapeutic agent for ischemic heart disease.

## Introduction

Ischemic heart disease (IHD) is a common disease that accounts for the major proportion of cardiovascular diseases, causing numerous deaths globally (Goff et al., 2014). It is generally known that cardiac circulation insufficiency is a primary risk factor, causing oxygen free radical production and myocardial energy metabolism disturbance (Chouchani et al., 2014; Kornfeld et al., 2015). Furthermore, the blood reperfusion inevitably leads to detrimental effects such as apoptosis-induced cardiomyocyte death (Hausenloy and Yellon, 2013). These consequences bring about mitochondrial dysfunction of myocardial cell, especially an abnormal mitochondrial dynamics, which are regulated due to two opposing processes, mitochondrial fission and fusion (Han et al., 2018). The dynamic balance of fusion and fission of mitochondria is essential in determining their morphology, number, subcellular distribution, and function. The core protein factors for mitochondrial fission and fusion are dynamin proteins that possess membrane-remodeling properties. A growing literature supports the role of abnormal fission and fusion in heart failure (Knowlton et al., 2014; Dorn, 2016). Although the current treatments for IHD could improve blood supply for the heart, it is still an intractable obstruction to completely restore the balance of mitochondrial dynamics in time. Thus, reversion of abnormal mitochondrial dynamics is a hard but significant step toward improving treatment for the patients.

In the past decade, mitochondrial fusion and fission imbalance has been one of the high-profile topics in cardiovascular diseases (Del Campo et al., 2018), neurodegenerative diseases (Ammal Kaidery and Thomas, 2018; Godoy et al., 2018), obesity (Knott and Bossy-Wetzel, 2010), etc. Mitochondria are highly dynamic organelles constantly going through fusion and fission (Kasahara et al., 2013). Mitochondrial fission is a divisive phenomenon in which a single mitochondrion separates into two or more mitochondria followed by fragmentation of mitochondria and rapidly high energy demand, and on the other hand, mitochondrial fusion is an inverse process, two or more mitochondria form into a single mitochondrion (Franco et al., 2016; Haroon and Vermulst, 2016). By consuming lots of energy (Piquereau et al., 2013), cardiomyocytes keep the balance of mitochondrion fusion and fission when facing to the suitable energy requirement and having favorable metabolic condition (Diaz and Moraes, 2008; Li and Liu, 2018). Growing individuals have emphasized the role of mitochondrial fusion and fission in the process of IHD and heart failure. Increased mitochondrial ﬁssion and decreased mitochondrial fusion in myocardial ischemia/reperfusion (I/R) injury will disturb vascular homeostasis leading to cardiomyocyte apoptosis (Karbowski et al., 2004; Ashrafian et al., 2010; Yang et al., 2017). Timothy Wai and his colleagues showed that imbalanced fusion protein optic atrophy 1 (Opa1) processing and mitochondrial fragmentation would cause heart failure in mice (Wai et al., 2015). Moreover, previous research revealed that upregulation of mammalian fission protein fission mitochondrial 1 (FIS-1) powerfully promoted apoptosis, while Opa1 might perform as an anti-apoptotic protein to keep spontaneous apoptosis in check (Lee et al., 2004). Therefore, maintaining favorable balance of mitochondrial fusion and fission or ameliorating excessive mitochondrial fission in IHD is essential for homeostasis of cardiomyocytes.

Lots of attention has been spent on the implications of antioxidants in cardiovascular disease (Zhang et al., 2008; Hatch et al., 2014). Unfortunately, clinical trials proved that the traditional antioxidants targeting reactive oxygen species (ROS) directly are in general ineffective and sometimes harmful in the context of cardiovascular pathology. The major reason for the failure of traditional antioxidants in the clinical studies is that ROS at lower levels exerts physiological effects by involving in signaling pathways while pathological ROS at excessive levels induces cell damage. Therefore, modulate molecular events upstream and downstream of ROS production, i.e., improve mitochondrial functions, may overcome the limitations of traditional antioxidants. Mitochondria-based therapeutics that alleviate mitochondrial dysfunction and maintain the balance of mitochondrial dynamics seem to be a promising therapeutic approach for myocardial ischemia and heart damage (Kornfeld et al., 2015).

Algae are capable of photosynthesis and have been used in traditional Chinese medicine for a variety of biological activities. The extracts of cyanobacteria, blue-green algae, have been found to show widespread pharmacological activities in treating wound healing (Gunes et al., 2017), neuroprotection (Lee et al., 2018), obesity (Seo et al., 2018), and inflammatory diseases (Gonzalez et al., 1999; Bermejo et al., 2008; Zahran and Emam, 2018). C-phycocyanin (C-pc) is one of the active components extracted and purified from blue-green algae, *Spirulina platensis* (Liu et al., 2016). Previous experiments found that C-phycocyanin (C-pc) served as a natural antioxidant (Remirez et al., 2002) and had anticancer (Jiang et al., 2018), anti-inflammatory, and anti-apoptotic activities (Romay et al., 2003). Studies have also showed that C-pc attenuated the formation of ROS and inhibited apoptosis in cardiomyocytes (Khan et al., 2006). However, whether C-pc affects mitochondrial dynamics or helps to balance abnormal mitochondrial fission and fusion in cardiomyocytes after I/R is unknown. Therefore, we used an oxygen–glucose deprivation/reoxygenation (OGD/R) model in H9c2 cells, an *in vitro* model to study the ischemia in the heart, to investigate the role of C-pc in mitochondrial fission and fusion dynamics. In the present study, we found that C-pc not only changed mitochondrial morphology from diminutive and globular to filamentous form but also decreased mitochondrial fission protein levels and elevated fusion protein levels, reversing excessive mitochondrial fission induced by OGD/R injury. Since excessive mitochondrial fission protein dynamin-like protein 1 (DLP-1) is triggered to cytochrome c release (Strack et al., 2013), while fusion protein Opa1 could prevent cytochrome c leakage from mitochondria (Frezza et al., 2006), our study showed that C-pc significantly decreased cytochrome c and apoptotic protease activating factor-1 (Apaf1) and inhibited the activation of procaspsae-9 in H9c2 cells induced by OGD/R. Furthermore, C-pc possibly exerted its effects by directly or indirectly modulating extracellular signal-regulated kinase 1/2 (ERK1/2) and c-Jun N-terminal protein kinase (JNK) pathway. Collectively, for the first time, we found that C-pc powerfully ameliorated excessive mitochondrial fission and promoted mitochondrial fusion, maintaining the balance of mitochondrial dynamics and protecting cardiomyocytes from intrinsic apoptosis. It might be a potential candidate agent against I/R injury of heart for further development.

## Materials and Methods

### Cell Culture

The myoblast cell line H9c2, derived from embryonic rat heart ventricle, was obtained from the Cell Bank of Chinese Academy of Sciences (Shanghai, China). Cells were cultured in a HERAcell 150i CO_2_-Incubator (Thermo Fisher Scientific Inc., USA) in Dulbecco’s modified Eagle’s medium (DMEM, Grand Island, NY, USA) supplemented with 10% fetal bovine serum and 1% penicillin/streptomycin (GIBCO-Invitrogen, Grand Island, NY) in a humidified incubator with 5% CO_2_/95% air atmosphere at 37°C.

### Induction of OGD/R and Treatment

For induction of I/R *in vitro*, H9c2 cells were exposed to hypoxic conditions (oxygen deprivation, 0.5% O_2_) for 24 h in a low-glucose DMEM medium (pH 7.4) containing 5.3 mM KCl, 44.0 mM NaHCO_3_, 110.3 mM NaCl, 0.9 mM NaH_2_PO_4_, and 1.8 mM CaCl_2_ at 37°C (oxygen–glucose deprivation). After hypoxia, the cells were reoxygenated and transferred to a normal culture condition (normoxic/normoglycemic) for 24 h at 37°C (reoxygenation).

**Figure 1:**
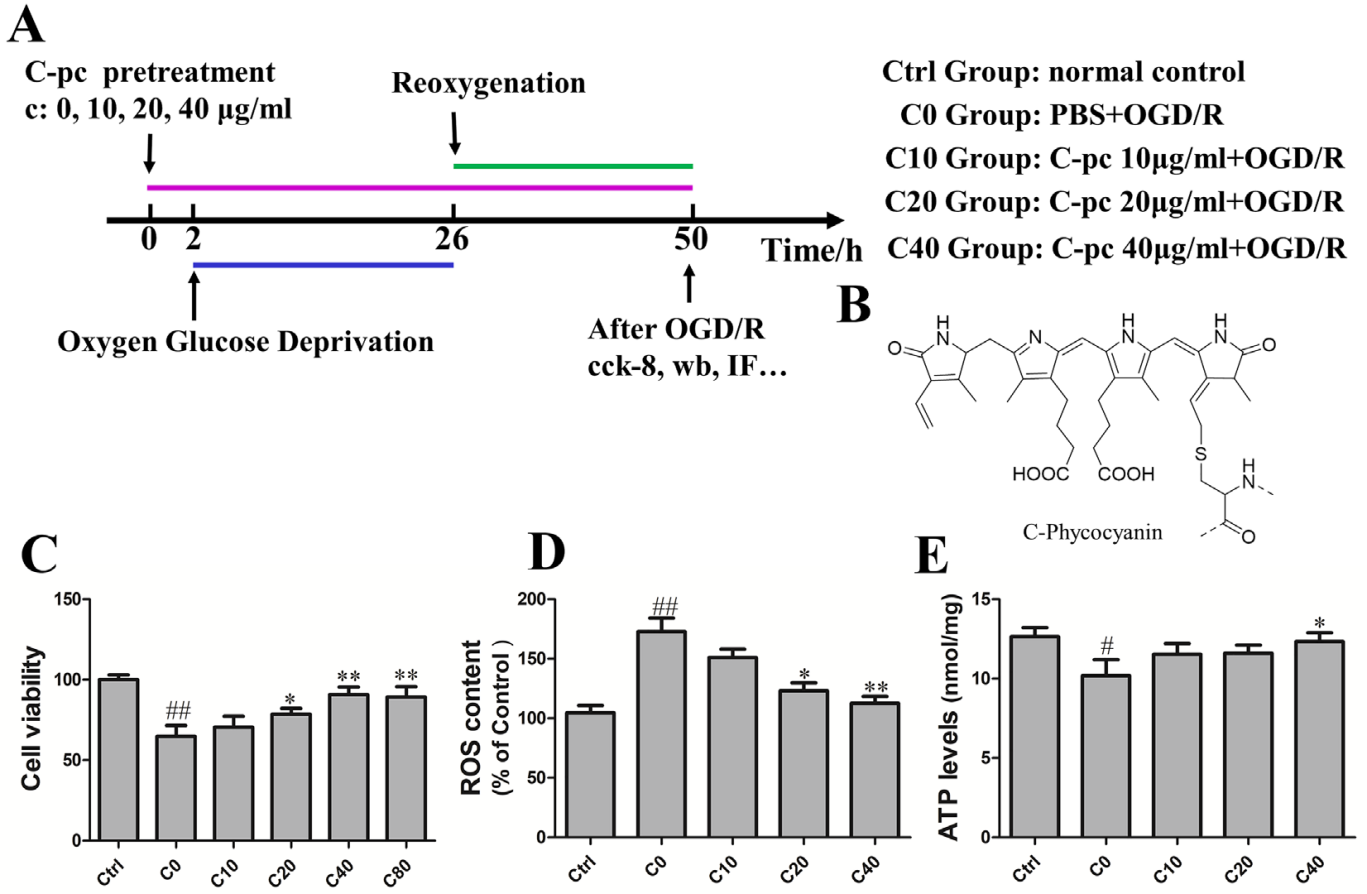
C-phycocyanin dose-dependently reduced oxygen–glucose deprivation/reoxygenation (OGD/R)-induced cell death and reactive oxygen species (ROS) in H9c2 cells. **(A)** Experiment design. The group C0 was exposed to OGD/R and treated with sterile phosphate-buffered saline (PBS). Groups C10, C20, and C40 were treated with C-phycocyanin (C-pc) at the concentration of 10, 20, and 40 μg/ml, respectively, in addition to the exposure to OGD/R. Cells in the control groups were cultivated under the normal conditions without exposing to OGD/R. **(B)** Chemical structure of the C-pc (Bharathiraja et al., 2016). **(C)** Effects of C-pc on OGD/R-induced cell death. Cell viability was detected by cell counting kit-8 (cck-8) assay. **(D)** Effects of C-pc on OGD/R-induced intracellular ROS. **(E)** Effects of C-pc on mitochondrial function assessed by an ATP detection kit. The ATP level was normalized by protein levels (per milligram per milliliter). Data were expressed as mean ± SEM (n = 5). ^#^ *P* < 0.05, ^##^ *P* < 0.01 versus control (normal) group; *P < 0.05, **P < 0.01 versus C0 (OGD/R model) group. *P* < 0.05 was considered as statistically significant.

To study the protective effect of C-pc, H9c2 cells were treated with C-pc (Sigma-Aldrich St. Louis, MO, USA) at different dosage (0, 10, 20, 40, and 80 μg/ml) 2 h before the hypoxia–oxygenation onset and lasted until the end of reoxygenation in C-pc-treated groups. The time schedule of the induction of OGD/R and C-pc treatment is shown in **Figure 1A**. The group C0 was exposed to OGD/R and treated with sterile phosphate-buffered saline (PBS). Groups C10, C20, and C40 were treated with C-pc at the concentration of 10, 20, and 40 μg/ml, respectively, in addition to the exposure to OGD/R. Cells in the control groups were cultivated under the normal conditions without exposing to OGD/R.

### Assessment of Cell Viability

Cell viability was assessed by cell counting kit-8 (cck-8) assays (Dojindo, Kumamoto, Japan). The H9c2 cells were seeded and cultured in a 96-well cell plate at 2 × 10^4^ cells/well for 24 h. Mediums with different dosages of C-pc (0, 10, 20, and 40 μg/ml) were used to treat cells for 24 h to detect the effect of C-pc. Afterward, cck-8 was added into the medium according to manufacturer’s instructions and incubated for 2 h. Finally, the absorbance value was measured at 490 nm with a microplate reader to manifest cell viability determination.

### Determination of Intracellular Reactive Oxygen Species Levels

Intracellular ROS levels were determined using dichloro-dihydro-fluorescein diacetate (Beyotime, China), which can cross the intracellular matrix where it is oxidized by ROS and produce fluorescent dichlorofluorescein. The H9c2 cells were seeded and cultured in 96-well cell plate at 2 × 10^4^ cells/well for 24 h. H9c2 cells were incubated with 10% dichloro-dihydro-fluorescein diacetate for 30 min at 37°C in a humidified incubator, and then, the plates were washed using DMEM without serum three times.

### Determination of Adenosine Triphosphate Levels

Adenosine triphosphate (ATP) levels were determined using an ATP detection kit (Beyotime, China). The H9c2 cells were seeded and cultured in a six-well cell plate at 2 × 10^4^ cells/well. The supernatant medium was substituted by PBS, and the cells were harvested using lysis buffer at 4°C. Then, lysis buffer was centrifuged at 12,000×g for 5 min, and obtained supernatant medium was intermingled with reagent. The data were analyzed by a multimode reader (Tecan M200, Switzerland). Finally, the ATP level was normalized by protein level (per milligram per milliliter).

### Western Blotting

H9c2 cells were harvested and washed with PBS for 3 min. Protein was extracted using radioimmunoprecipitation assay buffer with phenylmethylsulfonyl fluoride (1%, v/v) and protease/phosphatase inhibitor cocktails (Beyotime, Haimen, China). The concentrations of protein in samples were detected with bicinchoninic acid protein assay kit (Beyotime Biotechnology, Shanghai, China). Protein samples were separated by 8–15% sodium dodecyl sulfate polyacrylamide gel electrophoresis depending on the molecular weight of target protein and then transferred onto polyvinylidene difluoride membranes, blocked with 5% nonfat dry milk for 2 h at room temperature, incubated with specific primary antibodies at 4°C overnight (antibodies source was provided in **Supplementary material Table 1**). The following day, the polyvinylidene difluoride membrane was washed for three times with tris-buffered saline with Tween 20 (TBS-T) and incubated with the appropriate horseradish peroxidase secondary antibody for 2 h. After washing with tris-buffered saline with Tween 20 for three times, the blots were detected using an enhanced chemiluminescence (Millipore Billerica, USA) in multifunction imaging system (Tanon 5200 Multi, Shanghai, China).

### Immunocytochemistry

H9c2 cells were fixed with 4% paraformaldehyde in PBS for 15 min, permeabilized with 0.5% Triton X-100 for 15 min, then blocked with 10% goat serum for 1 h. Afterward, the cells were incubated with primary antibodies at 4°C overnight. The next day, the cells were washed with PBS and incubated with proper fluorescence-conjugated secondary antibodies (1:200 dilution) for 60 min, then washed with PBS, and the nuclei were counterstained with 4’,6-diamidino-2-phenylindole for 7 min. Images were taken using a fluorescent microscope (OLYMPUS DP72, Japan) and a confocal microscope (Leica TCS SP8, Germany).

### Staining of Mitochondria

H9c2 cells grew on coverslips inside a petri dish filled with DMEM. When cells reached the desired confluence, the media was removed from the dish and added with pre-warmed (37°C) staining solution containing mitochondrion-selective probe Mito-Tracker Red (MT-red) (Invitrogen, Carlsbad, CA, USA). The cells were co-stained with MT-red for 25 min under growth conditions. According to the procedures recommended by the manufacturer, the optimal concentration of MT-red was determined to 1 mM in preliminary experiments. After staining, cells were washed with fresh, pre-warmed DMEM and then fixed with freshly prepared, pre-warmed DMEM containing 2–4% formaldehyde. After fixation, H9c2 cells were incubated in PBS containing 0.2% Triton X-100 for 10 min and washed with PBS.

### Statistical Analysis

Results were represented as mean ± SEM. Comparisons involving more than two groups used one-way ANOVA followed by Fisher’s *post hoc* test. Comparisons of two groups used the two-tailed Student’s t test. *P* < 0.05 was considered as statistically significant.

## Results

### C-Phycocyanin Dose-Dependently Mitigated Cell Death, Reduced Reactive Oxygen Species, and Ameliorated Mitochondrial Function Induced by Oxygen–Glucose Deprivation/Reoxygenation in H9c2 Cells

During I/R, cardiomyocyte death attributed to the deterioration of heart damage. Cell viability was tested by cck-8 assay to determine the promising effect of C-pc on H9c2 cells subjected by OGD/R. As shown in **Figure 1C**, cell viability of H9c2 cells was significantly decreased in C0 group subjected by OGD/R compared with normal control group. In contrast, C-pc dose-dependently mitigated the cell death induced by OGD/R, as cell viabilities in C10, C20, and C40 groups increased with the concentration of C-pc. However, C-pc treatment at the dosage of 80 μg/ml was not more effective than that of 40 μg/ml. Trypan blue staining was also performed to determine the cell viability in case that cck-8 assay might be affected by mitochondrial function. The results of trypan blue staining and cck-8 were consistent. When treated with C-pc (40 μg/ml), there was obviously greater cell viability and fewer cell deaths in H9c2 cells subjected to OGD/R (data not shown). So, C-pc was used at the concentration from 0 to 40 μg/ml in later experiments.

Increased generation of ROS was suggested as a major contributor to the pathogenetic damage of I/R injury. We thus measured the levels of intracellular ROS. Compared with the normal control group, the ROS levels dramatically elevated in OGD/R treatment group. Similarly, the OGD/R-induced production of intracellular ROS was inhibited in C-pc-treated groups in a dose-dependent manner as well (**Figure 1D**). Moreover, the function of the mitochondrial was also assessed by the levels of ATP. C-pc at the dose of 40 μg/ml significantly enhanced the reduced ATP levels induced by OGD/R, improving OGD/R-induced inhibition of mitochondrial function (**Figure 1E**). Additionally, treatment with 40 μg/ml C-pc for 50 h did not significantly affect the cell viability, mitochondrial morphology, and ATP level of normal H9c2 cells (supplementary date).

### C-Phycocyanin Alleviated Mitochondrial Morphology Changes Induced by Oxygen–Glucose Deprivation/Reoxygenation

**Figure 2:**
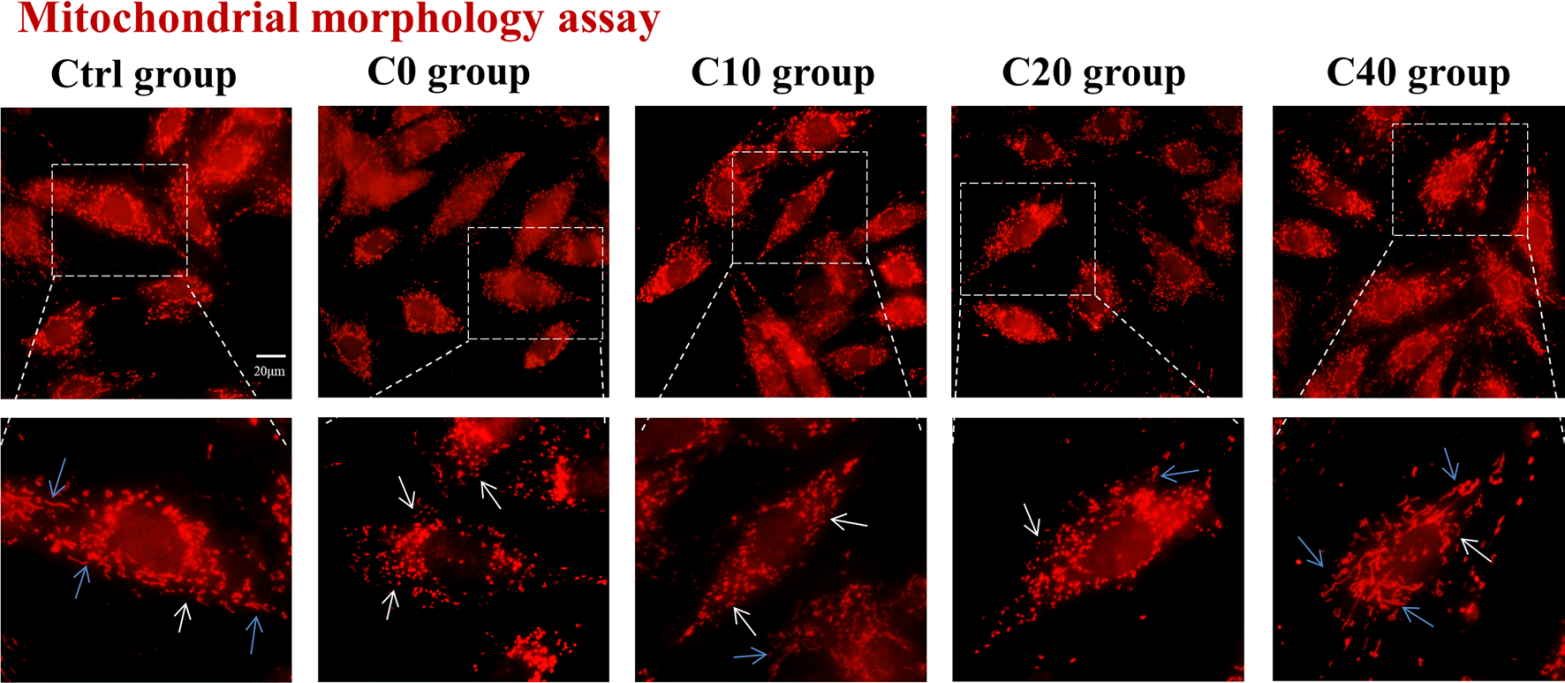
C-phycocyanin alleviated mitochondrial morphology changes induced by OGD/R. The mitochondrial morphology of H9c2 cells was visualized by mitochondrion-selective probe Mito-Tracker. Cells were treated with C-pc (0, 10, 20, and 40 μg/ml) or phosphate-buffered saline (PBS) and subjected to OGD/R except the control group. Representative images showed the morphology of mitochondria (scale bar 20 μm). The images in the dotted box are shown at a higher resolution on the below. The white arrow showed the diminutive and globular mitochondria, and the blue arrow showed filamentous mitochondria.

Mitochondria are dynamic organelles undergoing alteration of mitochondrial morphology of fusion and fission. The disruption of the balance between fission and fusion was one important source that led to the production of ROS and cell damage. To examine the effect of C-pc on the mitochondrial morphology of H9c2 cells subjected to OGD/R, the mitochondrion-selective probe MT-red was used to visualize mitochondria by fluorescence microscopy. In normal control cells, most mitochondria exhibited a filamentous structure as shown in **Figure 2**. Mitochondrial morphology in OGD/R-treated (C0) group differed from that in control cells and manifested as diminutive and globular shapes, suggesting excessive mitochondrial fission that occurred in H9c2 cells under the oxygen deficit and energy debt during OGD/R. The abnormal mitochondrial morphology was gradually improved with the administration of C-pc by increasing concentration, as the mitochondrial morphology had a general tendency toward filamentous mitochondria, which was the characterization of mitochondrial fusion and existed in normal H9c2 cells (**Figure 2**). Collectively, these results demonstrated that C-pc ameliorates mitochondrial morphology change that might be associated with mitochondrial fusion and fission balance in H9c2 cells after OGD/R.

### C-Phycocyanin Reduced the Expression of Mitochondrial Fission Proteins Dynamin-Like Protein 1 and Fission Mitochondrial 1, Suppressed Dynamin-Like Protein 1 Phosphorylation, and Transportation in H9c2 Cells Induced by Oxygen–Glucose Deprivation/Reoxygenation

**Figure 3:**
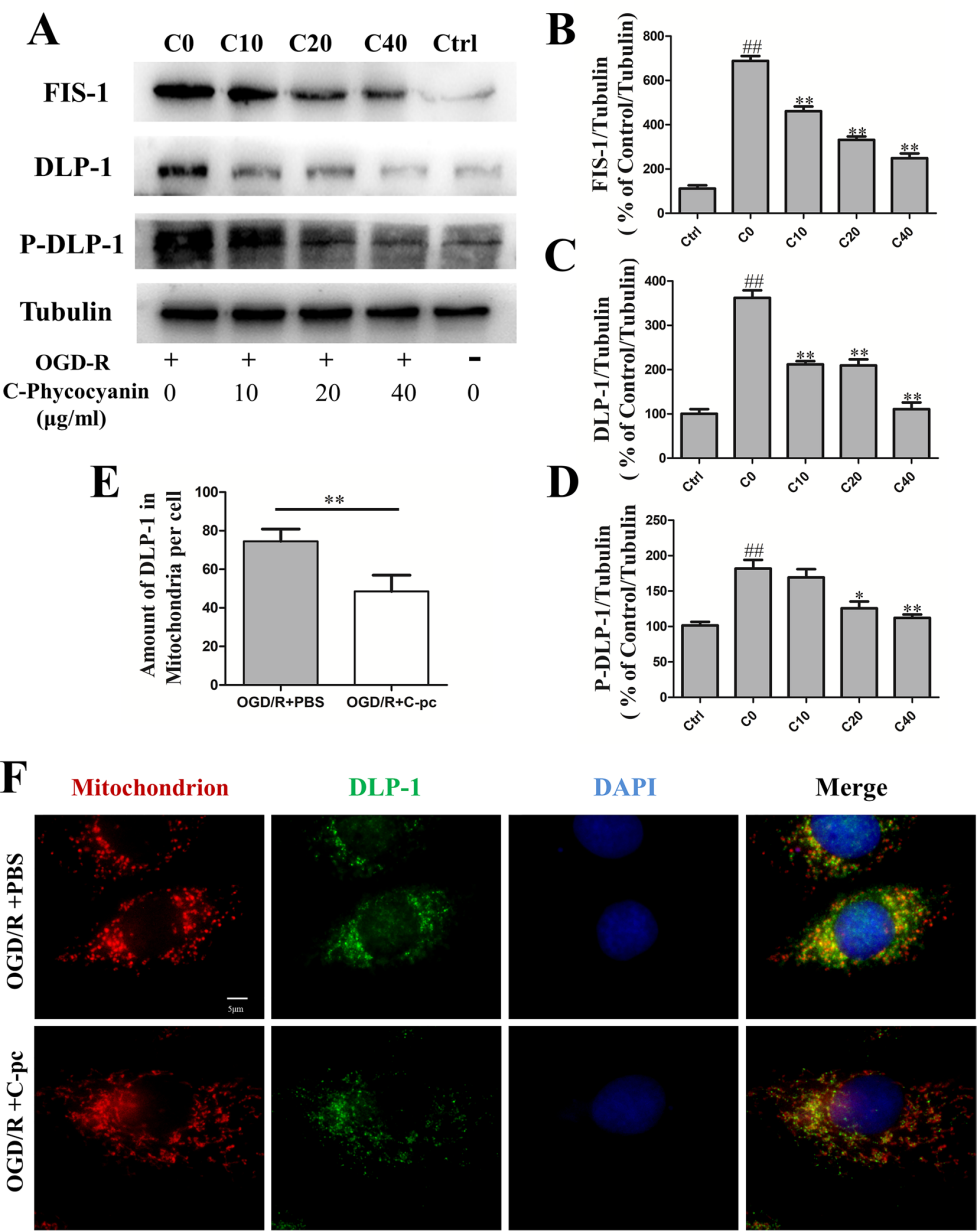
C-phycocyanin reduced the expression of mitochondrial fission proteins DLP-1 and Fis1, suppressed DLP-1 phosphorylation, and transportation in H9c2 cells induced by OGD/R. **(A)** Representative Western blot images show the expression of mitochondrial fission proteins (FIS-1 and DLP-1) and DLP-1 phosphorylation. **(B–D)** Densitometric quantitation is shown as percentage protein expression of FIS-1 **(B)**, DLP-1 **(C)**, and P-DLP-1 **(D)**. **(E)** Quantification analysis of the amount of DLP-1 in mitochondria (co-localization of DLP-1 and mitochondria). **(F)** Representative images from immunofluorescence staining of H9c2 cells treated with PBS or C-pc (40 μg/ml) to OGD/R exposure. The cells were immunostained with 4’,6-diamidino-2-phenylindole (blue), Mito-Tracker (red), and anti-DLP-1 antibody (green). Data were expressed as mean ± SEM (*n* = 5). ^#^ *P* < 0.05, ^##^ *P* < 0.01 versus control (normal) group; *P < 0.05, **P < 0.01 versus C0 (OGD/R model) group. *P* < 0.05 was considered as statistically significant.

A number of proteins, including DLP-1 and FIS-1, are involved in mitochondrial fission. Then, we detected the protein levels of DLP-1, DLP-1 phosphorylation, and FIS-1 by Western blot and DLP-1 transportation by immunocytochemistry. The levels of mitochondrial fission proteins, DLP-1 and FIS-1, remarkably increased in H9c2 cells that suffered from OGD/R compared with those in the normal control group (*P* < 0.01, **Figure 3A, B, and D**). The elevated levels of mitochondrial fission proteins induced by OGD/R were significantly reduced by the administration of C-pc in a dose-dependent manner (**Figure 3A, B, and D**). The expression of DLP-1 was even back to a near-normal level (**Figure 3D**). Phosphorylation of DLP-1 regulated the association of DLP-1 with mitochondria. To determine the effects of C-pc on DLP1-mediated fission, we examined the effect of C-pc on DLP-1 phosphorylation at serine 616 that has been shown to increase mitochondrial fission and found that C-pc treatment made a significant reduction in OGD/R-induced DLP-1 phosphorylation. In order to directly monitor the DLP-1 on mitochondrial surface, the localization and quantification of DLP-1 were further detected by immunocytochemistry. Consistent with the inhibitory effect in the expression of fission proteins induced by OGD/R, not only the total DLP-1, as indicated by weaker fluorescent intensity than that in OGD/R models (**Figure 3F**), but also the amount of DLP-1 on mitochondria, as the coexisted immunostaining of DLP-1 and mitochondria (**Figure 3F**), were remarkably decreased by the treatment of C-pc (40 μg/ml), as shown in **Figure 3E**, suggesting C-pc could mitigate OGD/R-impaired recruitment of DLP-1 from cytoplasm to mitochondrial surface, where DLP-1 could work effectively.

### C-Phycocyanin Promoted Mitochondrial Fusion Proteins Mitofusins 1 and 2 and Optic Atrophy 1 Expression

**Figure 4:**
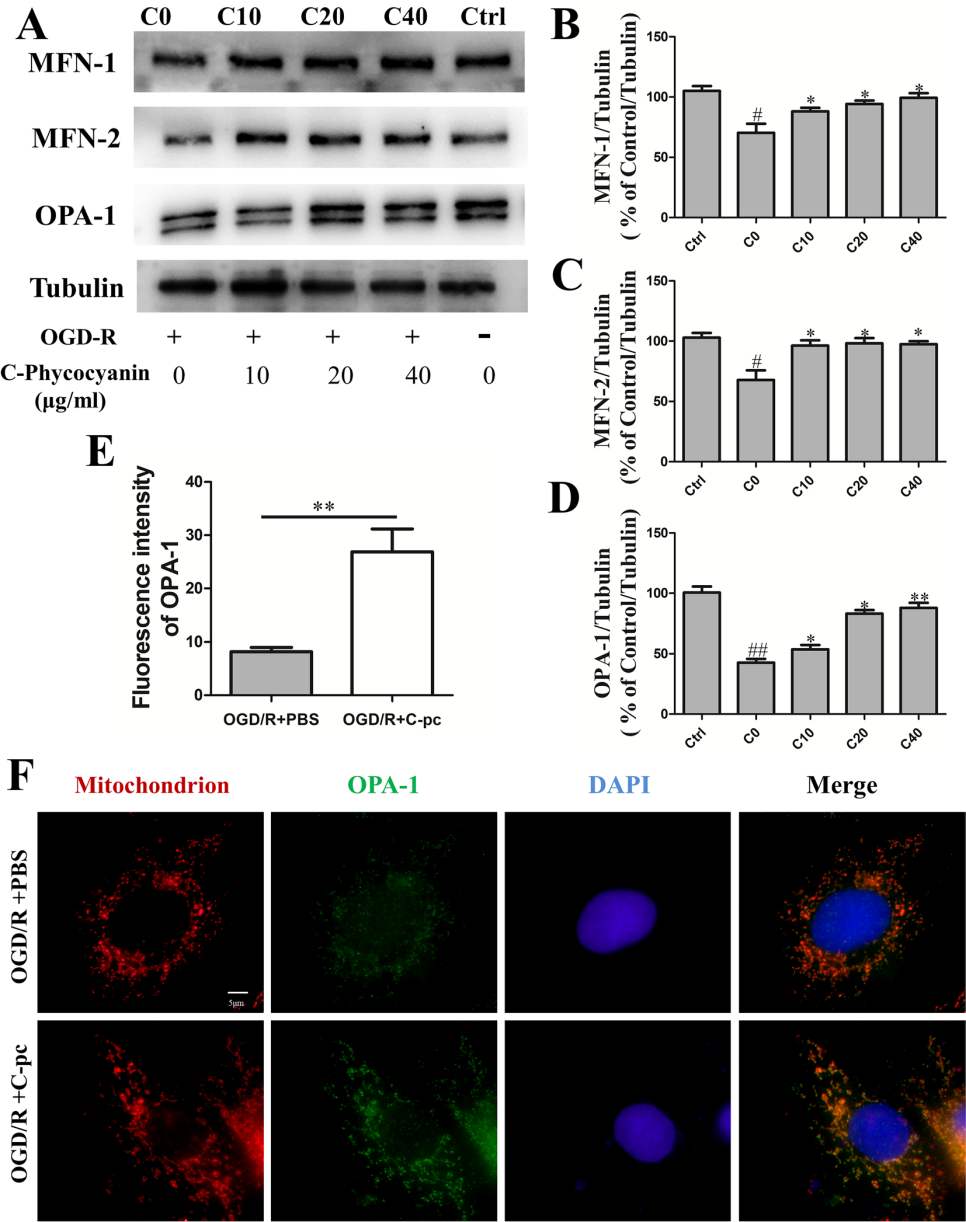
C-phycocyanin promoted mitochondrial fusion proteins Mfn1, Mfn2, and Opa1 expression. **(A)** Representative Western blot images show the expression of mitochondrial fusion proteins MFN-1, MFN-2, and OPA-1. **(B–D)** Densitometric quantitation is shown as percentage protein expression of mitochondrial outer membrane: MFN-1 expression **(B)**, MFN-2 expression **(C)**, and mitochondrial inner membrane OPA-1 expression **(D)**. Vertical coordinates used (protein/β-actin)/(control/β-actin). **(E)** Quantification analysis of the fluorescence intensity of OPA-1 on H9c2 cell treated with PBS or C-pc (40 μg/ml) after OGD/R exposure. **(F)** Representative images from immunofluorescence staining of H9c2 cells treated with PBS or C-pc (40 μg/ml) to OGD/R exposure. The cells were immunostained with 4’,6-diamidino-2-phenylindole (blue), Mito-Tracker (red), and anti-OPA-1 antibody (green). Data were expressed as mean ± SEM (*n* = 5). ^#^ *P* < 0.05, ^##^ *P* < 0.01 versus control (normal) group; *P < 0.05, **P < 0.01 versus C0 (OGD/R model) group. *P* < 0.05 was considered as statistically significant.

Mitochondrial fusion was confirmed to be regulated by fusion proteins including mitofusins (Mfn1 and Mfn2) and Opa1. We performed Western blot to investigate whether C-pc attenuated the inhibition of fusion proteins expression induced by OGD/R to keep the balance of mitochondrial dynamics. The results showed that outer mitochondrial membrane proteins Mfn1 and Mfn2 in the control group were observably higher than those in C0 group that suffered from OGD/R (**Figure 4B–C**, *P* < 0.05). Similarly, the level of inner mitochondrial membrane protein Opa1 had similar change (**Figure 4D**, *P* < 0.01). Additionally, the abnormal outer mitochondrial membranes of both Mfn1 and Mfn2 in C0 group were significantly improved when the cells were treated with a low-dose C-pc (10 μg/ml) and even came back to the level of the control group (**Figure 4B–C**). However, only when treated with much higher concentration of C-pc (40 μg/ml), the inner mitochondrial membrane protein Opa1 got back to the normal level as that in the control group. These results correlated with that OGD/R-induced damage on the inner membrane of the mitochondria that was more serious than that on the outer membrane in H9c2 cells (**Figure 4B–C**).

Meanwhile, we carried out immunofluorescence to determine the quantification of Opa1 in C0 and C40 groups. The same results were obtained with Western blot. Thus, these results indicate that C-pc potently prevents the decrease of fusion proteins Mfn1, Mfn2, and Opa1 expression induced by OGD/R.

### C-Phycocyanin Attenuated Intrinsic Apoptosis Induced by OGD/R Injury in H9c2 Cells

**Figure 5:**
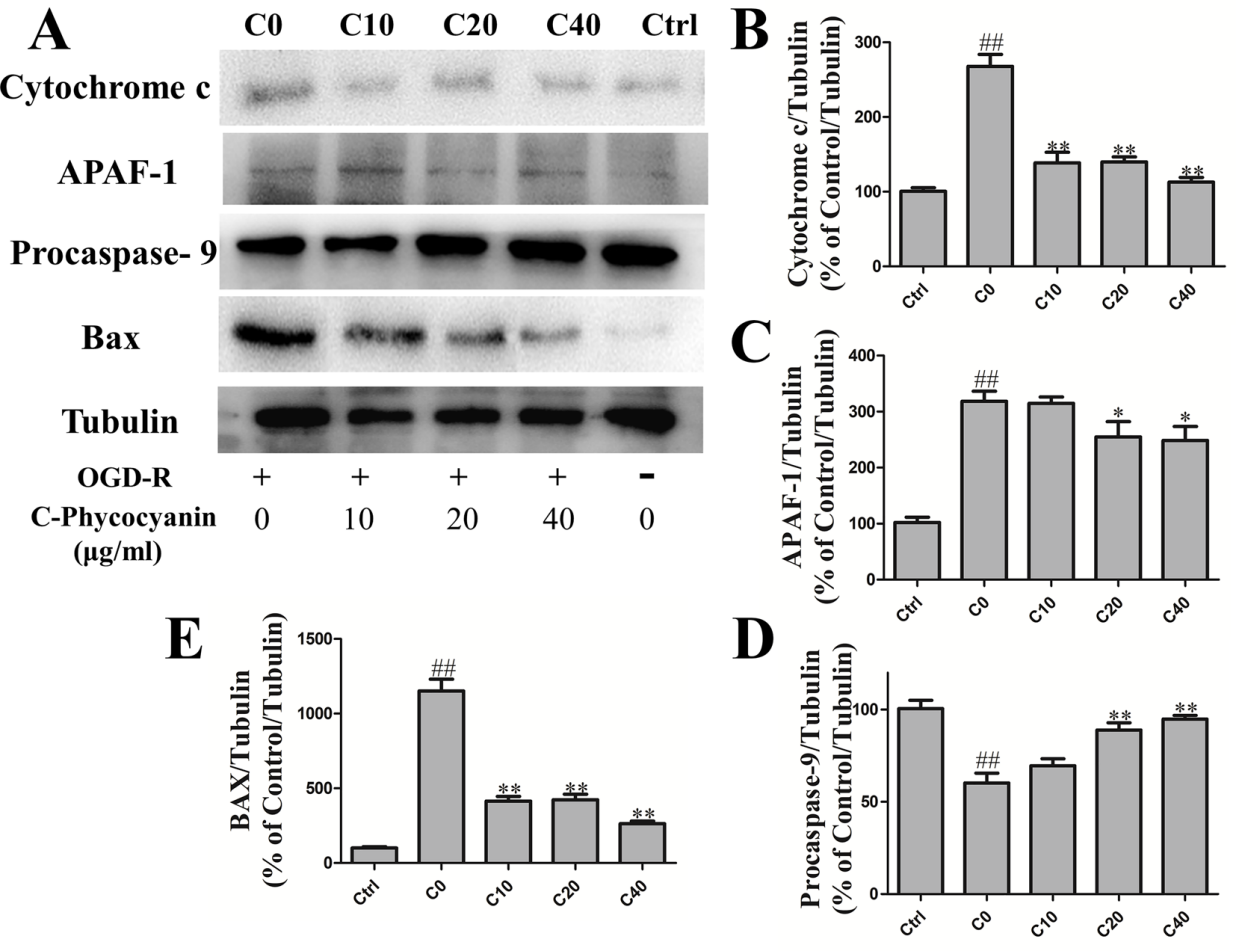
C-phycocyanin attenuated intrinsic apoptosis induced by OGD/R injury in H9c2 cells. **(A)** The Western blot of endogenous apoptosis pathway proteins and Bax. **(B–E)** The Western blotting of cytochrome c expression **(B)**, Apaf-1 **(C)** and procaspase-9 **(F)**, and Bax **(E)** in H9c2 cells. For detecting protein expression, cells were treated with C-pc (0, 10, 20, and 40 μg/ml) or PBS and subjected to OGD/R expect control group. Data were expressed as mean ± SEM (*n* = 5). ^#^ *P* < 0.05, ^##^ *P* < 0.01 versus control (normal) group; *P < 0.05, **P < 0.01 versus C0 (OGD/R model) group.

Apoptosis played a crucial role of cardiomyocyte death in IHD. Focusing on the mitochondria of H9c2 cells, we investigated whether C-pc could attenuate mitochondrial apoptosis induced by acute OGD/R injury in H9c2 cells. The Western blot results showed that under the OGD/R, there was a higher level of cytochrome c in the C0 group than the control group. Cytochrome c is the main factor of intrinsic apoptosis, which releases from mitochondria and associates with the complex of apoptotic protease activating factor-1 (Apaf1) and procaspase-9. As expected, the protein level of Apaf1 and activated procaspase-9 in the C0 group was significantly increased than that in the control group (**Figure 5B–D**). These results demonstrate the increase of H9c2 cells’ intrinsic apoptosis that occurred with OGD/R injury. In the treatment of C-pc (40 μg/ml), the protein level of cytochrome c and Apaf1 was significantly decreased, and the activation of procaspsae-9 was also inhibited compared with that of the C0 group (*P* < 0.01). In addition, H9c2 cells treated with C-pc exceedingly inhibited the Bax expression (**Figure 5E**).

### C-Phycocyanin Modulated Phosphorylation of Extracellular Signal-Regulated Kinase 1/2, c-Jun N-Terminal Protein Kinase Induced by Oxygen–Glucose Deprivation/Reoxygenation Injury in H9c2 Cells

**Figure 6:**
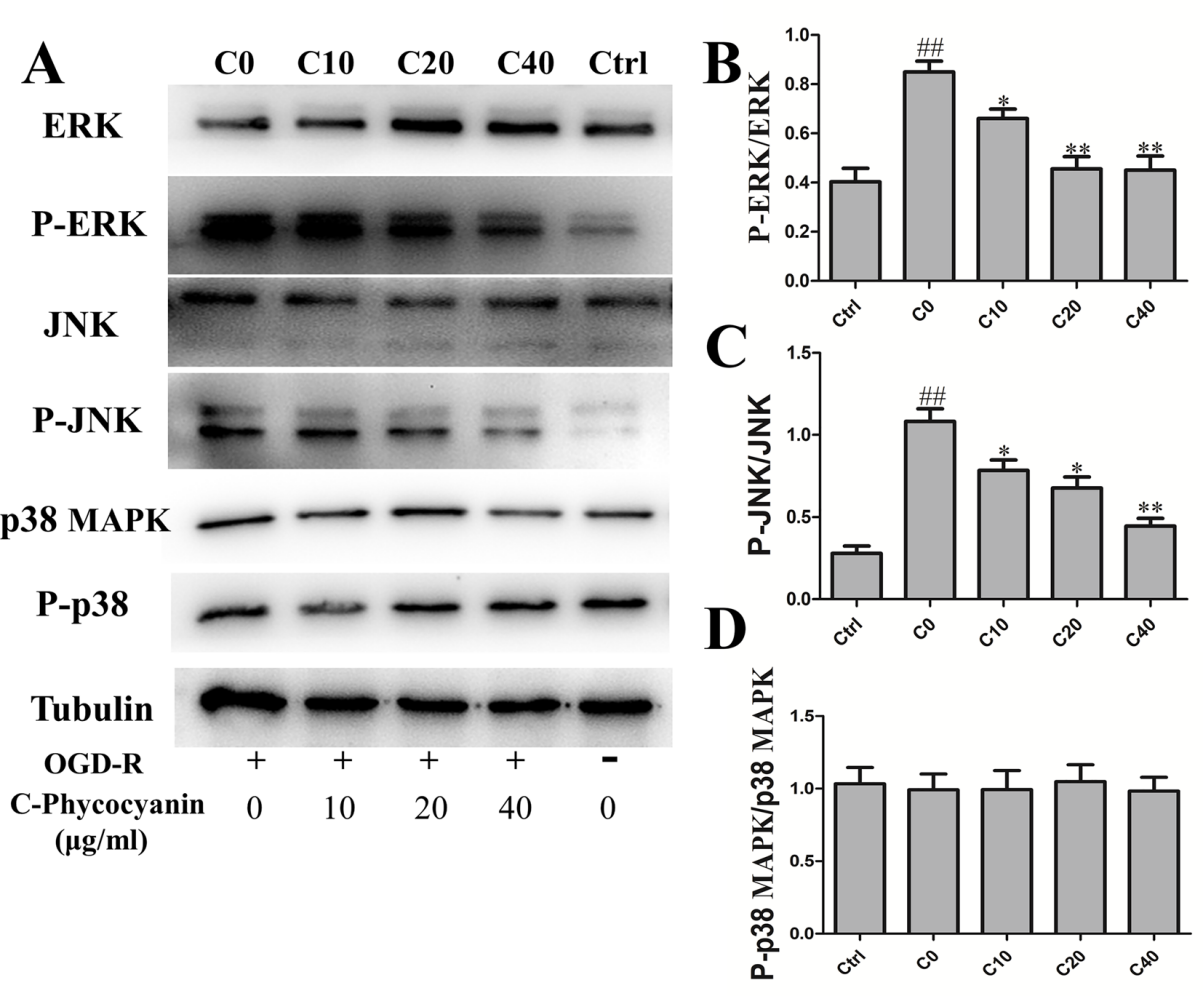
C-phycocyanin modulated phosphorylation of ERK 1/2, c-Jun N-terminal protein kinase (JNK) induced by OGD/R injury in H9c2 cells. **(A)** The WESTERN blotting of MAPK pathway. **(B–D)** The ratio of Western blotting results P-ERK/ERK **(B)**, P-JNK/JNK **(C)**, and P-p38 MAPK/p38 **(D)** expression in H9c2 cells. Vertical coordinates used phosphorylation/non-phosphorylation. Data were expressed as mean ± SEM (*n* = 5). ^#^ *P* < 0.05, ^##^ *P* < 0.01 versus control (normal) group; *P < 0.05, **P < 0.01 versus C0 (OGD/R model) group. *P* < 0.05 was considered as statistically significant.

Mitogen-activated protein kinases (MAPKs) were involved in various fundamental cellular processes, including proliferation, differentiation, apoptosis, and OGD/R injury. The Western blot was used to detect the levels of ERK1/2, JNK, p38 MAPK, and phosphorylation after H9c2 cells suffered from OGD/R. The results showed that the phosphorylation of ERK1/2 was obviously elevated in the C0 group (twofold over control **Figure 6A**), which could be attenuated by treatment with C-pc. Furthermore, a significant increase in the activation of JNK was observed in the C0 group compared with that in the control group (7.39-fold over control **Figure 6C**). C-pc modulated phosphorylation of JNK with the same trend of ERK1/2. However, OGD/R treatment seemed to have no effect on p38 MAPK, and C-pc also could not influence p38 MAPK signaling pathway (**Figure 6D**).

## Discussion

Based on the cardioprotective effects of C-pc, we paid attention to the effects on mitochondrial dynamics and used it to treat H9c2 cells in OGD/R model. Fortunately, we found that C-pc effectively inhibited ROS production and kept the balance of mitochondrial dynamics. Additionally, the decrease of excessive mitochondrial fission inhibited mitochondrial apoptosis *via* cytochrome c/Apaf1 pathway. These protective effects of C-pc play an essential role in preventing cardiomyocytes from the death triggered by OGD/R *in vitro*.

Cardiomyocytes consume lots of energy, in which 90% of ATP is produced by mitochondria (Li and Liu, 2018). The complicated and subtle balance of mitochondrial fusion and fission should be in no doubt that it is crucial for cardiomyocyte homeostasis. Previous research has suggested that Opa1 mutant and Mfn2 knockout mice displayed significantly greater mitochondrial dysfunction than those in the control group inducing cardiomyocyte death (Chen et al., 2012; Zhao et al., 2012). Further, decreased DLP-1 expression in cardiomyocytes could enhance myocardial contractility, while inhibition of Mfn2 could decrease contractility (Givvimani et al., 2015). These results indicate that gene defect or mutant of fusion protein accelerates cardiomyocyte death, and mitochondrial fission is not beneficial for ischemic myocardial protection. Excessive fission proteins contribute to mitochondrial fragmentation, which can induce a metabolic switch from fatty acid to glucose utilization and generate massive mitochondria, consuming plentiful energy in the heart (Wai et al., 2015). However, it almost stores no spare energy in sustained ischemic condition. In other words, mitochondrial fusion represents a recognized strategy to allow survival during nutrient deprivation and cellular stress (Gomes et al., 2011). The observed fragmentation might be the result of DLP-1-mediated fission and of impaired mitochondrial fusion (Karbowski et al., 2004). Here, our work highlights the importance of the balance of mitochondrial dynamics, and C-pc can inhibit excessive mitochondrial fission induced by OGD/R in cardiomyocyte.

Apoptosis has been recognized as one of the major factors of cardiomyocyte death induced by I/R. It has been studied that C-pc could exert anticancer effects *via* upregulation of Fas and cleaved caspase-3 protein levels, while downregulation of the Bcl-2 protein level in MDA-MB-231 cells to induce apoptosis (Jiang et al., 2018). Conversely, C-pc played a protective effect in cardiomyocytes by upregulating anti-apoptotic protein (Khan et al., 2006). Also, inhibition of oxidative stress by C-pc prevented cisplatin-induced nephrotoxicity (Fernandez-Rojas et al., 2014). Considering that C-pc treatment decreases ROS production, affects mitochondrial dynamics greatly, and improves cardiomyocyte survival, it is possible that C-pc can mediate its protective action in cardiomyocytes by suppressing intrinsic apoptosis that is less known before. Earlier studies have demonstrated that ischemic and hypoxic stage caused cardiomyocyte apoptosis that were associated with mitochondrial fission and fusion (Ong et al., 2010; Ikeda et al., 2015). On the one hand, Opa1 can regulate cristae shape, and Mfns can inhibit mitochondrial outer membrane permeabilization preventing cytochrome c, a proapoptotic factor, leakage (Frezza et al., 2006; Chen et al., 2009). On the other hand, inhibition of mitochondrial fission can increase inner membrane proton leak that contributes to decrease ROS production and susceptibility to apoptotic stimuli (Lee and Yoon, 2014; Wang et al., 2015). To further investigate this possibility, H9c2 cells were synchronized and subjected by OGD/R for 48 h. Finally, we observed that C-pc significantly reduced cytochrome c release from mitochondria and inhibited the activation of endogenous apoptotic pathways (**Figure 5A**). H9c2 cells are commonly used to generate oxygen–glucose deprivation–nutrition resumption (OGD/R) models to simulate myocardial I/R injury *in vitro*, as they are derived from heart tissue. However, we should also be careful about using H9c2 cells in the present study, as Apaf-1, a molecule in the pathway of apoptosis, was expressed in H9c2 cells but not cardiomyocytes, which might make H9c2 cells more sensitive to apoptosis than cardiomyocytes (Sanchis et al., 2003). Thus, more research on the cardioprotective effects of C-pc in cardiomyocytes *in vivo* is needed for ensuring its potential use in treatment of IHD.

The molecular weight of monomeric C-pc is about 40 kD, which is an obstruction for entering into cytoplasm directly to exert protective action. Mitogen-activated protein kinases (MAPKs) are involved in various fundamental cellular processes, including cell apoptosis and survival. We speculate that whether these enzymes are activated through a sequential phosphorylation cascade that affects fission and fusion protein expression and apoptosis by C-pc directly or indirectly. Previous studies have shown that extracellular signal-regulated kinase (ERK) is well-documented for apoptosis induced by DNA-damaging agents (Fehrenbacher et al., 2008) and various antitumor compounds (Kim et al., 2008). Further, on the presence of ROS, sustained ERK can be activated (Cagnol and Chambard, 2010). Our results show that ERK is activated in H9c2 induced by OGD/R and C-pc can significantly suppress ERK activity. The ERK activity is associated with intrinsic apoptotic pathway and ROS production leading to cells death, while the inhibition of ERK activity by C-pc modulates its participation in mitochondrial dynamics. Pyakurel et al. showed that the activation of ERK phosphorylated Mfn1 causing the inhibition of mitochondrial fusion and stimulation of apoptotic mitochondrial permeabilization (Pyakurel et al., 2015). Furthermore, activation of ERK can accelerate phosphorylation of DLP-1, which leads to mitochondrial fragmentation and facilitates apoptosis (Strack et al., 2013). The c-Jun N-terminal protein kinase (JNK) also plays an important role in the regulation of cell apoptosis (Weng et al., 2016). Here, we demonstrated that the JNK activity significantly increased in response to OGD/R in H9c2. Indeed, C-pc can inhibit the phosphorylation of ERK1/2 and JNK and improve cardiomyocyte survival.

**Figure 7:**
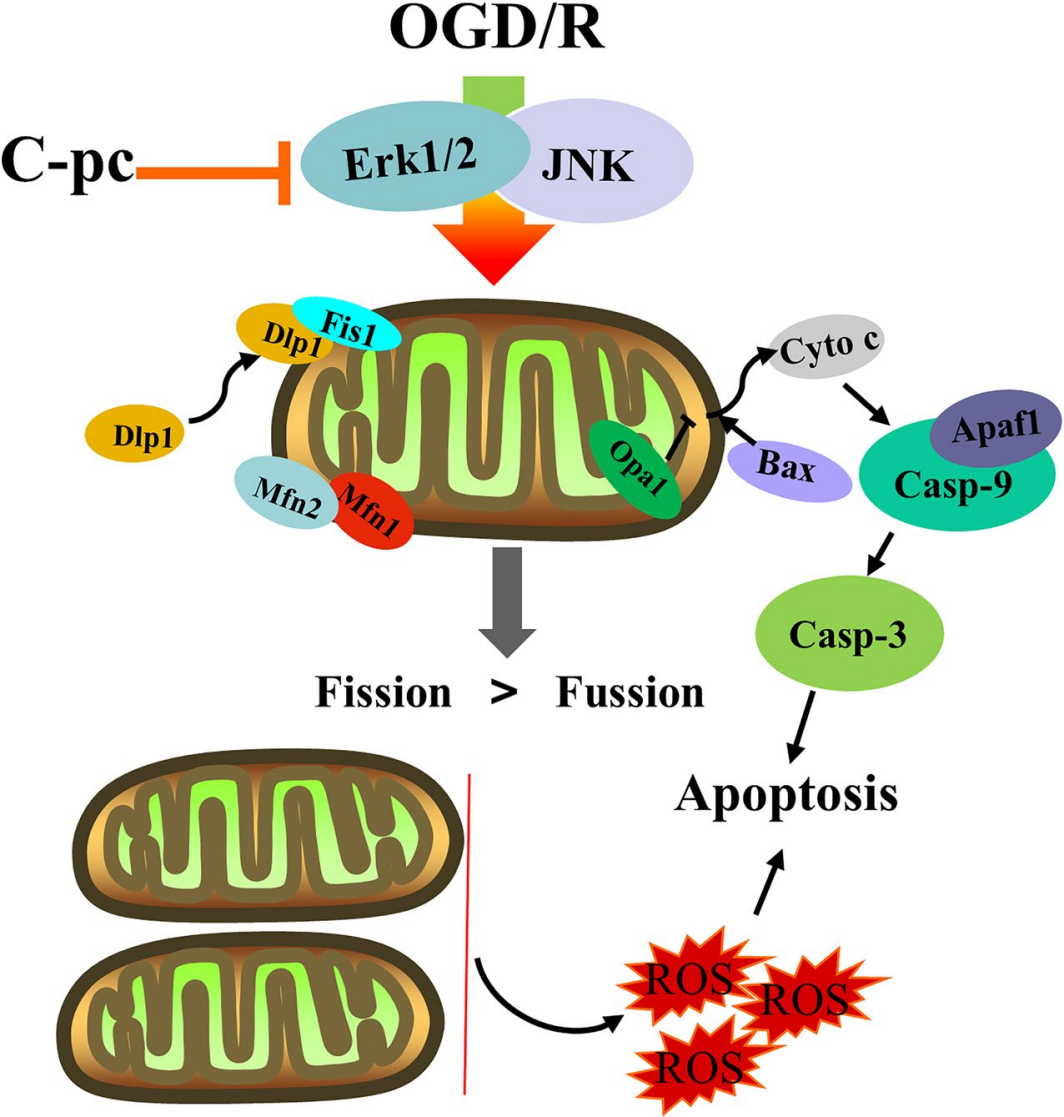
The effects of C-phycocyanin in H9c2 cells that suffered from OGD/R injury. C-pc decreases the unnormal mitochondrial dynamics by suppressing the expression of fission proteins and promoting the expression of fusion proteins by extracellular inhibition of ERK1/2 and JNK. C-pc also inhibits Dlp-1 phosphorylation and the recruitment of Dlp1 from cytoplasm to mitochondrial surface. Further, the effect of C-pc on inhibition of excessive mitochondrial fission ameliorates mitochondrial apoptosis *via* cytochrome c/Apaf1 pathway.

In conclusion, our results suggest that C-pc can keep the balance of mitochondrial dynamics and suppress mitochondrial apoptosis *via* cytochrome c/Apaf1 pathway induced by OGD/R (**Figure 7**). C-pc might be a new therapeutic alternative or potential cardioprotectant to ameliorate excessive mitochondrial fission in IHD.

## Author Contributions

WZ and JH contributed to the design of the study, served as the study coordinators, and edited the manuscript. JG designed the study, performed experiments, collected and analyzed the data, and wrote the manuscript. LZ performed experiments and collected and analyzed the data. JW, LZ, DZ, JQ, HW, and MY helped perform experiments and analyzed data. All authors have read and approved the final manuscript.

## Funding

This study was supported by grants from the National Natural Science Foundation of China (grant no. 81471232, 81570293) and the Science and Technology Commission of Shanghai Municipality (14431901400).

## Conflict of Interest Statement

The authors declare that the research was conducted in the absence of any commercial or financial relationships that could be construed as a potential conflict of interest.

The handling editor declared a shared affiliation, though no other collaboration, with several of the authors JG, LZ, WZ, MY, and HW at time of review.
