# Supplementary material for: C-Phycocyanin Ameliorates Mitochondrial Fission and Fusion Dynamics in Ischemic Cardiomyocyte Damage
Source: Front Pharmacol. 2019 Jun 28;10:733. doi: 10.3389/fphar.2019.00733 (PMC6611522; doi:10.3389/fphar.2019.00733)
Supplement: Supplementary file 1 [file Table_1.docx]

| REAGENT or RESOURCE | SOURCE | IDENTIFIER |
| --- | --- | --- |
| Antibodies |  |  |
| Mouse monoclonal anti-DLP1 | BD Biosciences | Cat#611112 |
| Mouse monoclonal anti-OPA1 | BD Biosciences | Cat#612606 |
| Rabbit monoclonal anti-MFN2(D2D10) | Cell Signaling Technology | Cat#9482 |
| Rabbit polyclonal anti-MFN1 | Proteintech | Cat#13798-1-AP |
| Rabbit polyclonal anti-FIS1 | Proteintech | Cat#10956-1-AP |
| Rabbit polyclonal anti-BAX | Cell Signaling Technology | Cat#2774 |
| Rabbit polyclonal anti-APAF1 | Proteintech | Cat#21710-1-AP |
| Rabbit polyclonal anti-p44/42 MAPK (Erk1/2) | Cell Signaling Technology | Cat#9102 |
| Rabbit polyclonal anti-Phospho-p44/42 MAPK (Erk1/2) | Cell Signaling Technology | Cat#9101 |
| Rabbit monoclonal anti-p38 MAPK (D13E1) | Cell Signaling Technology | Cat#8690 |
| Rabbit monoclonal anti-Phospho-p38 MAPK (D3F9) | Cell Signaling Technology | Cat#4511 |
| Rabbit polyclonal anti-SAPK/JNK | Cell Signaling Technology | Cat#9252 |
| Mouse monoclonal anti- Phospho -SAPK/JNK (G9) | Cell Signaling Technology | Cat#9255 |
| Rabbit monoclonal anti- Cytochrome c (D18C7) | Cell Signaling Technology | Cat#11940 |
| Mouse monoclonal anti- Caspase-9 (C9) | Cell Signaling Technology | Cat#9508 |
| Rabbit polyclonalβ-Actin | SAB Signalway Antibody | Cat#21338-2 |
| Goat anti-Mouse igG HRP | SAB Signalway Antibody | Cat#L3032-2 |
| Goat anti-Rabbit igG HRP | SAB Signalway Antibody | Cat#L3012-2 |
| Goat Anti-Mouse IgG 488-conjugated Affinipure | Proteintech | Cat#SA00006-1 |

| Biological cell Chemicals, Proteins, and Detection kit | | SOURCE | IDENTIFIER |
| --- | --- | --- | --- |
| H9C2 | Cardiac myoblast | | Cell Bank of Chinese Academy of Sciences |

| C-Phycocyanin (CPC) | Sigma | Cat#P2172 |
| --- | --- | --- |

**Mitochondrion-selective probes Invitrogen**  **Cat#M7512**

| Cell counting kit-8 (cck-8) | Dojindo | Cat#CK04 |
| --- | --- | --- |
| Reactive oxygen species assay kit | **Beyotime** | **Cat#S0033** |
